# Annotations

**Published:** 1900-12-29

**Authors:** 


### Annotations.

### International Tabulation of Causes of Death.

For some years past efforts have been made to bring the methods adopted in different countries in regard to the tabulation of the causes of death into greater accord than exists at present. This is a matter of much interest and importance. Great as are the political differences between different countries, the *rapprochement* in regard to all matters of science is one of the most marked features of recent years. In some sciences, indeed, it is essential, and in none more so than in that which concerns vital statistics. Such statistics lose all their value unless they are comparable, and this they cannot be unless the basis on which they are drawn up is the same in every country. It is to be hoped, then, that a general and wide recognition will be given to the labours of the International Statistical Congress of 1900 in regard to the tabulation of causes of death. But behind the very important question of the tabulation of statistics lies that of the collection thereof, and it is to be confessed that until England can make a better show in regard to the registration of the causes of death, all this talk about uniform tabulation and international classification carries with it a strong dash of unreality. It would seem a small thing, in a civilised country, to ask that means should be taken to ascertain and to record the cause of every death which takes place; and we have no doubt that most people, being aware of the existence of a system of registration, with its registrars of births and deaths in every village, imagine that such is the case in England. But nothing could be further from the truth. We have registrars, it is true, but so long as they are allowed to accept any tale that is told them as to the cause of death, and so long as the permitted laxity in this respect is such as to lead to the registration of ten thousand deaths annually under the head of "uncertified," we cannot but feel that questions of international statistics are somewhat premature.

### Tuberculous Meat.

In warring against a disease so fatal and widespread as is tuberculosis one not unnaturally desires to eradicate every possible means of infection, and it having been ascertained, as we think without any possibility of doubt, that feeding upon the flesh of tuberculous animals is in certain cases an efficient cause of tuberculosis, there is a strong feeling among some in favour of entirely prohibiting the sale of such meat for the food of man; and no doubt such a course would be strictly logical. Nevertheless it would be vastly expensive, seeing that according to the computation of experts on the subject it would condemn at least 40 per cent. of the cattle in this country as unfit for food. In practice, then, the question takes this form: Can we find a compromise? And, if so, where shall we draw the line? According to the extremists, the answer to these questions really depends upon whether or not tuberculosis can ever be regarded as a purely localised affection. But even this is a question which will be very differently answered according to the sort of lesion which we admit to constitute such a localised tuberculosis. Whichever way one looks at the matter, then, one is driven to the conclusion that a compromise of one sort or another must be accepted, and, for the present at least, there can be but little doubt that the medical officers of health throughout the country must be content to go by the rules laid down by the Local Government Board last year in its "Instructions to Meat Inspectors with regard to tuberculosis." These instructions are liable to revision. We can have no doubt that their enforcement for a few years will have the effect of leading farmers to be more careful in the treatment of their live stock, and thus to a very considerable diminution in the prevalence of tuberculosis among cattle; and, needless to say, when that shall have happened the main objection to more stringent rules will have been removed, and then it will be possible to advance another step. In the meantime, however, we cannot but feel that the practice of selling the carcasses of tuberculous animals as prime beef is wrong, and even dishonest, and that it is much to be regretted that such a practice should receive the sanction given to it by the Local Government Board. If the butcher who buys a good-looking beast which on slaughter is discovered to contain a certain limited amount of tubercle is given the benefit of the doubt, and is allowed to dispose of the meat, he surely should not be permitted to sell it as prime beef. There are different ways of cooking, some of which are no doubt destructive to the bacillus tuberculosis. But some ways of cooking are certainly no protection whatever. Although, in view of the dearness of food, we will not offhand quarrel with the sale of the good and apparently healthy parts of the carcasses of beasts only locally affected, as permitted in the "Instructions," we are sure that some means should be taken to mark such meat, and thus give warning as to its origin. Meat of this kind need not be thrown away, but it should be properly cooked, and certainly ought not to be eaten in the form of an underdone steak. The sanction given by the Local Government Board to its free and unrestricted sale as prime beef is, we think, a mistake which ought to be rectified as soon as possible.

### Water Filtration in America.

It is somewhat interesting to observe that, notwithstanding all the hard things which have been said in regard to the quality of the water supplied to London, and notwithstanding the many schemes which have been put forward from time to time with the object of obtaining a better water from sources undefiled, the people of New York are now quoting London as an example, and the London water as a proof of the efficacy of filtration as a means of manufacturing a pure drinking-water from sources which are known to have been contaminated. New York has long been in doubt about its water-supply, and lately, in consequence of the many complaints which have been made, a complete inquiry into the whole subject has been undertaken. As a result the conclusion has been arrived at that the only effective and permanent means by which a remedy can be applied to existing evils consists in the adoption of an efficient system of filtration. The cost will be great, but we can hardly doubt that not only New York, but most of the American towns, will have, before long, to provide themselves with artificially purified drinking-water. In a new country it was right enough to trust to such water as was supplied by nature. The towns were far apart, water was plentiful, and mostly it was free from all chances of human contamination. But America has long since ceased to be a new country, typhoid fever has so increased in prevalence as to be regarded as constantly endemic in many parts, and we need not be surprised that many of its towns are looking to art rather than to nature, and to the example of old countries rather than of new in their efforts to obtain pure drinking-water.

